# The Feasibility of Increasing Hospital Surge Capacity in Disasters through Early Patient Discharge

**DOI:** 10.29252/beat-070203

**Published:** 2019-04

**Authors:** Sima Feizolahzadeh, Aliakbar Vaezi, Ali Taheriniya, Masoud Mirzaei, Mohammadreza Vafaeenasab, Davoud Khorasani-Zavareh

**Affiliations:** 1 *Department of Emergency and Disaster Health, Shahid Sadoughi University of Medical Sciences, Yazd, Iran*; 2 *Department of Nursing, School of Nursing and Midwifery, Research Center for Nursing and Midwifery Care in Family Health, Shahid Sadughi University of Medical Science, Yazd Iran*; 3 *Department of Emergency* *Medicine, Alborz University of Medical Sciences, Karaj, Iran*; 4 *Research Center of Prevention and Epidemiology of Non-Communicable Disease, Shahid Sadoughi University of Medical Sciences, Yazd, Iran *; 5 *Yazd Cardiovascular Research Centre, Shahid Sadoughi University of Medical Sciences, Yazd, Iran *; 6 *Safety Promotion and Injury Prevention Research Center, Shahid Beheshti University of Medical Sciences, Tehran, Iran*

**Keywords:** Early discharge, Hospital surge capacity, Disaster

## Abstract

**Objective:**

Hospitals are expected to be able to provide quality services during disasters. However, hospital capacity is limited and most hospital beds are almost always occupied. The aim of this study was to determine the feasibility of increasing hospital surge capacity during disasters through identification of patients suitable for safe early discharge.

**Methods:**

This cross-sectional study was conducted from May 2017 to February 2018 in two phases. In phase I, the Early Discharge Checklist was developed by a multidisciplinary panel of experts. Then in phase II, the checklist was used to assess the dischargeability of 396 in-patients in general wards of hospitals in Alborz province, Iran. Data were analyzed through the SPSS software (v. 22.0) and the results were presented by descriptive and analytical statics at a significance level of less than 0.05.

**Results:**

Of 396 patients, (64.65%) were male, (68.9%) were married, and (38.6%) aged more than 54. Moreover, (34.6%) patients were dischargeable. Patients in cardiology wards were more dischargeable. At follow-up assessment, 33.3% of patients had been discharged after 48 hours. There was a significant relationship between patient dischargeability and 48-hour hospitalization status (*p*=0.001). Dischargeability had no significant relationships with patients’ demographic characteristics (*p*>0.05).

**Conclusion:**

A considerable percentage of in-patients are dischargeable during disasters. The Early Discharge Assessment Checklist, developed in this study, is an appropriate tool to provide reliable data about early dischargeability in disasters.

## Introduction

Health protection is the first concern of human beings during disasters. Thus, the number of people who refer to hospital settings unexpectedly and suddenly increases immediately after disasters, so that hospital management may be impaired [1]. The ability to provide quality of care proportionate to patient surge has always been among the main concerns of healthcare delivery systems during disasters[2]. Management of massive patient surge during disasters necessitates abundant resources and activities [3-6]. However, hospital resources are usually inadequate for fulfilling the numerous needs of a large number of disaster victims. For instance, hospital capacity is mostly limited and there are few empty beds even in normal conditions. Thus, strategies are needed to increase hospital surge capacity (HSC) during disasters [3, 7-9]. HSC is the ability of a hospital in rapidly expanding and promoting its services during disasters or mass casualty incidences [4, 10, 11]. The four main elements of HSC include stuff, staff, structure, and system. Thus, HSC can be increased through discharging patients with no serious conditions, withholding routine activities, elevating staffing level, using vacant spaces, and increasing medication and equipment supplies [12, 13].

Early patient discharge is one of the methods for increasing HSC [14, 15]. Through this method, patients who will not be at serious health risks after treatment discontinuation are discharged [4, 16]. A study into the emergency department response capacity in crises in Iran reported that early discharge of patients with stable conditions can increase emergency department capacity by 27.5% [17]. Another study in Iran reported that using different capacity-increasing measures increased the admission capacity of the emergency department from 16 to 42 patients[18]. 

Similarly, a study in the United States reported that one third of hospitalized patients were dischargeable within 24 hours [10]. A series of studies by Kelen et al. in the United States also showed that hospitalized patients can be categorized according to their dischargeability into five levels ranging from absolutely non-dischargeable to dischargeable with no serious complications [3]. Around 44% of hospitalized patients were then determined as dischargeable because they did not need critical care [19]. Moreover, 11% of hospitalized patients were identified as immediately dischargeable and 13% of them as dischargeable within 96 hours [16]. A study in Norway used the results reported by Kelen et al. and reported a 16%-increase in bed surge capacity within four hours [20].

Despite the known importance of early discharge to HSC, there are no clear guidelines for identifying dischargeable patients [10, 16, 19, 21, 22]. Moreover, there is limited information about dischargeable patients and HSC increase following early patient discharge. Thus, the present study was designed and carried out to determine the feasibility of increasing HSC during disasters through early patient discharge.

## Materials and Methods

This cross-sectional analytical study was conducted from May 2017 to February 2018 in two main phases. The first phase was related to the development of the Early Discharge Assessment Checklist (EDAC) and the second phase was related to the use of the checklist to assess early discharge among a sample of hospitalized patients.

 *Phase I: Development of the EDAC*

The criteria for early discharge were developed based on the comments of a multidisciplinary panel of experts (ten males and three females) who were interested in disaster management. All experts were faculty members of Alborz University of Medical Sciences, Alborz, Iran, and were working in hospitals affiliated to the university. The means of their age and work experience were 42 and 16, respectively. We held several sessions with the experts, from May to September 2017, in order to generate the checklist items. In the first session, the aims and necessity of the study and a summary of the existing literature were provided to the experts. Moreover, a hypothetical scenario was presented to them respecting a disaster in a neighbor province which would result in the transfer of its victims to the hospitals in Alborz province, Iran. The assumptions related to the hypothetical disaster scenario and the necessity of early patient discharge were as the following:

- The Emergency Operation Center of the local university announces the disaster in a neighbor province and requires the maximum level of alert;
- Around 80% of all hospital beds are occupied;
- There is an overcrowding in the hospital due to the transfer of the disaster victims;
- This critical situation is expected to continue at least for 72 hours;
-  The quality of care should be maintained up to standard levels.

As the aim of the study was to estimate the feasibility of early discharge, rather than the actual discharge of patients, the experts were asked to determine the basic criteria for early discharge in case of disasters which would create the need for increasing HSC. Accordingly, the experts agreed on 25 essential items on early discharge. The items fell into the four main domains of abnormal vital signs (six items), serious symptoms or conditions (nine items), the need for in-hospital medical interventions (six items), and abnormal laboratory findings (four items). The primary checklist was piloted in a teaching hospital in Karaj, Iran, and the findings were presented to the experts. They amended the items based on the findings and agreed on the final checklist which included 25 items in the following four domains:

- Vital signs: a temperature of less than 36°C or more than 38.5°C; a blood pressure of less than 90/60 or more than 180/11 mm Hg; a pulse rate of more than 100 beats per minute; a respiratory rate of more than 22 per minute; a Glasgow Coma Scale score of less than 15; and an arterial oxygen saturation of less than 95%;
- Serious symptoms or conditions: any neurologic symptoms in the past 48 hours; gastrointestinal bleeding in the past 48 hours; nausea/vomiting after eating; uncontrolled diabetes mellitus; acute asthma; chronic obstructive pulmonary disease; mental disorders associated with the risk of harm to the self or others; acute coronary syndrome; and convulsions in the past 24 hours;
- The need for in-hospital medical interventions: emergency surgery; relocation of joint dislocation; intravenous antibiotic therapy; advanced wound care (with debridement); cardiac monitoring; and respiratory support;
- Abnormal laboratory findings: a hemoglobin level of less than 8 mg/dL; a blood sodium level of less than 130 mEq/L; a blood potassium level of more than 5.5 mEq/L; and a blood glucose level of less than 80 or more than 250 mg/dL.

Items were rated either “Yes” (“Present”) or “No” (“Absent”). If all items were rated “No”, the intended patient was considered dischargeable. However, even one item with “Yes” response showed that the intended patient was non-dischargeable. Early discharge assessment for each patient was started using the vital signs items and continued with the items in the serious symptoms or conditions, the need for in-hospital medical interventions, and the abnormal laboratory findings.

The content validity of the checklist was assessed using content validity ratio and index. For content validity ratio calculation, ten experts in different medical specialties rated the necessity of each checklist item as either “Essential”, “Useful but unessential”, and “Unessential”. On the other hand, for content validity index calculation, the same experts rated the simplicity, relevance, and clarity of each item on a four-point scale. All items had content validity ratios and indices of respectively more than 0.62 and 0.8 and hence, none of them were deleted [23].

 *Phase II: Early discharge assessment*

 *Study setting*

 The study was conducted in teaching and non-teaching hospitals affiliated to Alborz University of Medical Sciences, Karaj, the center of Alborz province, Iran. Alborz province is located twenty kilometers west to Tehran, the capital of Iran, and has six counties with a total population of 2712400 people. This province is the neighbor of three provinces with high disaster rate (i.e. Tehran, Qazvin, and Gilan) and is located in the road of more than fifteen provinces in Iran. Karaj, the center of the province, is the fourth most populated city in Iran with a population of 1615218 people[24]. There are eleven public hospitals in the province, including four teaching and seven non-teaching hospitals, all of which are affiliated to Alborz University of Medical Sciences, Karaj, Iran. At the time of the study, three hospitals had a low bed occupation rate and one hospital was a maternity hospital. These four hospitals were not included in the study. 

Finally, seven hospitals were studied, which included three teaching and four non-teaching hospitals (Table 1). All 396 patients who were hospitalized in the general wards of these hospitals during the study were recruited to the study through census. Based on the comments of the experts who participated in checklist development, patients in pediatric, psychiatric, and burn care wards, coronary care units, and pediatric, neonatal, and adult intensive care units were not included in the study due to their special care needs.

**Table 1:** Characteristics of the studied hospitals

**Characteristics** **Hospitals**	**Hospital type**	**Wards**	**Beds**	**Patients**
**Shahid Bahonar**	Teaching	4	61	51
**Shahid Rajaei**	Teaching	10	154	79
**Shahid Madani**	Teaching	7	207	144
**Sarallah**	Non-teaching	2	27	16
**Imam Jafar Sadegh (PBUH)**	Non-teaching	2	76	48
**Shariati**	Non-teaching	2	86	42
**Imam Hasan (PBUH)**	Non-teaching	2	38	16
**Total**		29	649	396

*Data collection*

 Necessary data were collected using a demographic and clinical characteristics questionnaire and EDAC. The questionnaire contained items on patients’ age, gender, marital status, educational level, hospitalization ward, length of hospital stay, and medical diagnosis based on the tenth edition of the International Classification of Diseases [25]. This questionnaire also included one item on patient hospitalization status at 48 hours after early discharge assessment. The two possible responses to this item were “Has discharged” and “Still hospitalized”. The second data collection instrument was EDAC, which assessed patient dischargeability using 25 items in the four aforementioned domains. For data collection in each hospital, one day of the weak was randomly selected using a table of random numbers. Then, we referred to the intended hospital in the selected day and started data collection. We continued data collection in that hospital until all patients in its general wards were selected and assessed. Data collection was performed from October 2017 to February 2018. Each patient’s hospitalization status was re-assessed 48 hours after early discharge assessment.

 *Data analysis*

 The collected data were analyzed using the SPSS software (v. 22.0). Results were presented using the measures of descriptive statistics such as mean, standard deviation, frequency, and percentage. The relationships of dischargeability with patients’ demographic characteristics were assessed through the Chi-square or the independent-sample *t* tests. The independent-sample *t* test was also used to compare dischargeable and non-dischargeable patients respecting the length of their hospital stay on the data collection day. All statistical analyses were performed at a significance level of less than 0.05.

 *Ethical considerations*

 This study was approved by Shahid Sadoughi University of Medical Sciences, Yazd, Iran (with the code of IR.SSU.SPH.REC.1395.129). Necessary permissions for the study were also obtained from Alborz University of Medical Sciences, Karaj, Iran. All patients were ensured about the confidentiality of their data and their informed consents were obtained.

## Results

In total, 396 patients hospitalized in general hospital wards were studied. They were mostly male (64.65%) and married (69.94%) and more than one third of them had below-diploma education (34.6%) and aged more than 54 (38.64%) (Table 2). Moreover, around half of the patients were hospitalized in general surgery wards (49.75%) and the most common health problem among them was musculoskeletal disorders (24.75%).

**Table 2:** The relationships of patients’ characteristics with early dischargeability

** Dischargeability** **Characteristics**		**Dischargeable** **(N = 137)a**	**Non-dischargeable** **(N = 259)**	***P*** ** valueb**
		**N (%)**	**N (%)**	
**Age (Years)**	14–24	17 (37.8)	28 (62.2)	0.871
	25–34	25 (32.1)	53 (67.9)	
	35–44	29 (38.7)	46 (61.3)	
	45–54	16 (35.6)	29 (64.4)	
	≥ 55	50 (32.7)	103 (67.3)	
**Gender**	Male	82 (32)	174 (68)	0.147
	Female	55 (39.3)	85 (60.7)	
**Marital status**	Single	30 (32.6)	62 (67.4)	0.843
	Married	97 (35.5)	176 (64.5)	
	Other	10 (32.3)	21 (67.7)	
**Educational status**	Illiterate	16 (27.6)	42 (72.4)	0.07
	Guidance school	39 (28.5)	98 (71.5)	
	High school	36 (39.5)	55 (60.5)	
	University	46 (41.8)	64 (51.8)	
**Length of stay(Days)**		3.61±3.4	5.5±7.4	0.001

a : Percentage values have been rounded and hence, total values may not be equal to 100

b : The results of the Chi-square or the independent-sample *t* tests

Among 396 studied patients, 137 were dischargeable (34.6%) and 259 were non-dischargeable (65.4%). The means of hospital stay among all patients and among dischargeable and non-dischargeable patients were 4.8±6.3, 3.5±3.4, and 5.5±7.4 days, respectively. The mean of hospital stay among dischargeable patients was significantly less than their non-dischargeable counterparts (P<0.001). At 48-hour follow-up assessment, 132 patients had been discharged (33.3%), while 264 patients were still hospitalized (66.7%). The Chi-square test showed significant relationship between patient dischargeability and 48-hour hospitalization status (P=0.001). In other words, initial assessment revealed that one third of patients were dischargeable and 48-hour follow-up assessment revealed that one third of patients had been discharged. The same statistical test also indicated a significant relationship between dischargeability and hospital ward, so that patients in cardiology wards were more dischargeable while patients in orthopedic wards were less dischargeable (P=0.005; Table 3). However, dischargeability had no significant relationships with patients’ characteristics (Table 2).

**Table 3:** Early dischargeability and its relationship with hospital ward

**Dischargeability** **Wards**	**Dischargeable**	**Non-dischargeable**	***P*** ** valuea**
	**N (%)**	**N (%)**	0.005
**Internal medicine**	21 (38.9)	33 (61.1)	
**Infectious diseases**	6 (27.3)	16 (72.7)	
**Orthopedic**	12 (18.2)	54 (81.8)	
**General surgery**	73 (37.1)	124 (62.9)	
**Cardiology**	16 (59.3)	11 (40.7)	
**Neurology**	9 (30)	21 (70)	
**Total**	137 (34.6)	259 (65.4)	–––

a : The results of the Chi-square test

## Discussion

In this study, a checklist for early discharge assessment, called EDAC, was developed by a panel of experts for the first time in Iran. Then, the checklist was used to identify dischargeable patients and assess the feasibility of increasing HSC. It is important to note that EDAC is a short and simple-to-use instrument with simple Yes/No items which assesses patient dischargeability in the four main domains of abnormal vital signs, serious symptoms or conditions, the need for in-hospital medical interventions, and abnormal laboratory findings. The main findings of the present study were the early dischargeability of around one third of patients who were hospitalized in general hospital wards and the actual discharge of one third of them within 48 hours after our initial assessment.

Study findings showed that 34.6% of hospitalized patients in general hospital wards were dischargeable during a hypothetical disaster. It means that while there are many patients waiting for empty beds in normal conditions, an HSC of more than one third of the total hospital capacity can be created through early patient discharge. In line with our finding, an earlier study in the United States reported that one third of hospitalized patients were dischargeable within 24 hours [10]. Another study in the United States into the creation of HSC through early patient discharge reported that 44% of hospitalized patients did not need critical care and hence, were dischargeable [19]. Similarly, a study in Iran indicated that the early transfer of patients with stable conditions from the emergency department to other hospital wards increased the capacity of the emergency department by 27.5% [17]. Compared with hospitalized patients, disaster victims have greater need for hospital services; thus, early discharge of a large number of patients can significantly reduce mortality rate in disasters.

The dischargeability of around one third of the hospitalized patients in the present study implies that there is no necessity for the hospitalization of some patients. Unnecessary hospitalization of some patients is due to different factors such as delays in the process of medical consultations, physicians’ uncertainty about patient management, delays in performing some medical procedures, and unavailability of some equipment for diagnostic and paraclinical studies such as computed tomography scanning and magnetic resonance imaging [26]. The presence of medical science students in teaching hospitals also contributes to unnecessary patient hospitalization because some patients are kept hospitalized to teach their underlying conditions to students [27]. A study in Iran reported that medical factors, paraclinical factors, and hospital type (teaching or non-teaching) can affect length of stay in hospital [28]. Another studies in the United States also revealed that there is unnecessary hospitalization which is contributed to the following factors: presence of medical residents instead of medical specialists, postponement of procedures, and difficulty finding a bed in a skilled nursing facility [27, 29]. These findings highlight the necessity of developing strategies for managing factors behind unnecessary patient hospitalization, as well as a system approach on the phenomena that have been already shown in other studies [30]. Subsequent low bed occupation rate in hospitals before disasters paves the way for better management of disaster victims. More focus on educational plan can also fill this gap, is it shown in other related studies [31, 32].

The other finding of the present study was that 48 hours after initial dischargeability assessment using EDAC, one third of patients had been discharged from hospital. This finding confirms the reliability of the data obtained through EDAC. In other words, in line with EDAC data which revealed that one third of patients were dischargeable, 48-hour follow-up assessment indicated that one third of patients had been actually dischargeable. The availability of quality out-patient and home-based care services can provide the opportunity for early discharge of a large number of patients in [3, 19, 29] According to Hogg, implying hospital-based cares at home can reduce hospital bed occupancy in disasters [33]. 

 *Limitations and strengths of the study*

 A limitation of the study was the exclusion of patients in critical care units and pediatric, psychiatric, and maternity wards from the study. Therefore, more studies need to be conducted to estimate dischargeability or transferability of patients in critical care units in order to create surge capacity in these units. In this study there were no patient discharged practically, so future studies should be carried out on real discharge of low risk patients in controlled conditions and tracking for any untoward events. The strength of the study was the early discharge assessment of all patients in general wards in different hospitals, which expanded the generalizability of the study findings to other hospitals in other cities.

In conclusion, the current study shows the feasibility of early discharge of one third of patients hospitalized in general hospital wards in order to increase hospital surge capacity during disasters. The actual discharge of one third of patients within 48 hours after initial assessment highlights that EDAC can provide reliable data regarding the dischargeability of patients in general hospital wards. Implication of appropriate methods such as those introduced in this study for identification of low risk patients can help the decision makers in health system to estimate available beds in disasters. Ultimately, early discharge can significantly increase hospital capacity without any needs to develop other resources.
